# Miscellaneous Medical Intelligence

**Published:** 1850-05

**Authors:** 


### ARTICLE VII.

### MISCELLANEOUS MEDICAL INTELLIGENCE.

Dr. S. H. Dickson, Prof. of Theory and Practice in the University of the City of New-York, has resigned his Chair on account of ill-health.—His place is to be filled from applications made by the first of May inst. (Short notice).

It is rumored that Prof. Chapman, of the University of Pennsylvania, has resigned his Chair in that Institution.

We observe by the papers that a new Medical School is being organized at Milwaukee, Wisconsin.

Prof. Webster has been convicted of the murder of Dr. Parkman, and sentenced to be hung, but the day of execution has not yet been designated. The reward of $3,000, offered for Dr. Parkman's body, was paid to Mr. Littlefield on 6th of April.

A Bill has been presented in the Legislature of Mississippi, requiring Physician's prescriptions to be written in English instead of Latin.

The Cholera lingers about New-Orleans, and occasionally breaks out on board of boats on their passage up the Mississippi River and its tributaries; but it does not seem to be spreading with as much rapidity as last spring.

Prof. W. L. Attee has performed his seventh operation for Extripation of Ovarian Tumors.

It is announced in the Boston Medical and Surgical Journal, that Dr.'s Forbes and Marshall Hall, are about to visit this country.

It is said that chewing a piece of orange peel just before taking cod-liver oil, masks its taste entirely.

"Yankee Doctors.—Let Sir James Graham's Medical Bill come into operation, and Doctors will spring up among us as they do in America.— "Do you know what your Doctor was two years ago?" asked a Yankee not six months since of an Englishman, in Boston. "Do you know, stranger, what he was?" "No!" "Well, then, he was only a book-binder, I guess." At this news, the Englishman's jaw fell, and he returned silent thanks that his book-binder Doctor had not put him *in boards*."

Dr. G. W. Mayberry, of Alabama, says that blood externally applied is a most soothing and efficacious remedy in Erysipelas and burns.

A *Female* Medical School was to have been opened in Boston some time in April past.

Dr. Landon C. Rives, formerly Professor of Midwifery, etc., in Cincinnati College, has been appointed Professor of Materia Medica in the Medical College of Ohio, the Chair recently occupied by the late lamented Dr. Harrison.

Prof. J. K. Mitchell, of Jefferson Medical College, Philadelphia, in his late Valedictory Lecture, contends that the supply of Educated Physicians in the United States, is by no means equal to the demand.

A Belgian tribunal has decided that all professional visits by Physicians made between 9 o'clock P. M. and 6 o'clock A. M., are *night visits.*

Miss Dix, the eminent Philanthropist, has succeeded in persuading the Mississppi Legislature to build a Hospital for the Insane. An appropriation of $50,000 was made for the purpose.

